# The Role of Toll-like Receptors (TLRs) Mediated Inflammation in Pancreatic Cancer Pathophysiology

**DOI:** 10.3390/ijms222312743

**Published:** 2021-11-25

**Authors:** Arturo Orlacchio, Pellegrino Mazzone

**Affiliations:** 1 NYU Grossman School of Medicine, NYU Langone Health, New York, NY 10016, USA; 2 Biogem Scarl, Istituto di Ricerche Genetiche Gaetano Salvatore, 83031 Ariano Irpino, Italy

**Keywords:** pancreatic cancer, toll-like receptor, inflammation, chemotherapy

## Abstract

Pancreatic cancer (PC) is one of the most lethal forms of cancer, characterized by its aggressiveness and metastatic potential. Despite significant improvements in PC treatment and management, the complexity of the molecular pathways underlying its development has severely limited the available therapeutic opportunities. Toll-like receptors (TLRs) play a pivotal role in inflammation and immune response, as they are involved in pathogen-associated molecular patterns (PAMPs) and danger-associated molecular patterns (DAMPs). Activation of TLRs initiates a signaling cascade, which in turn, leads to the transcription of several genes involved in inflammation and anti-microbial defense. TLRs are also deregulated in several cancers and can be used as prognostic markers and potential targets for cancer-targeted therapy. In this review we discuss the current knowledge about the role of TLRs in PC progression, focusing on the available TLRs-targeting compounds and their possible use in PC therapy.

## 1. Introduction

Pancreatic cancer (PC) is a lethal malignancy with a high mortality rate that is projected to become the second leading cause of cancer death in the next ten years [1]. Based on the GLOBOCAN 2020, it has been estimated that pancreatic cancer causes more than 466,000 deaths per year worldwide, ranking as the seventh cause of cancer death in males and females [2]. Because of the lack of early diagnosis, about 80% of patients show unresectable tumor or metastases with a 5-year survival rate of about 10%. This parameter can increase to 58% in a small percentage of patients in which tumor is detected at early stages [3]. The standard treatment for patients affected by PC is surgical resection followed by chemotherapy. This strategy, supported by different studies, results in the improvement of survival outcome. In particular, the CONKO-001 study shows that the addition of gemcitabine treatment after tumor resection results in an increased 5-years survival rate with a slight increase in the overall survival [4]. Other clinical trials are carried out in order to identify the best therapeutic regime that improves patient survival. For example, dual treatment with capecitabine and gemcitabine after tumor resection results in the amelioration of the median overall survival [5]. Another therapeutic approach that is often applied to PC patients is the administration of FOLFIRINOX (5FU, leucovorin, irinotecan and oxaliplatin). This latter strategy appears to lead to a higher overall survival, progression-free survival and response rate when compared to gemcitabine single treatment [6]. However, due to its toxicity, this therapeutic regime is not suitable for all patients making gemcitabine the standard drug used in the PC treatment [6]. Despite the advances in the understanding of pancreatic cancer pathogenesis, the causes of the insurgence of this neoplasia still remain unknown. Environmental factors, such as smoking, obesity, diabetes mellitus and chronic pancreatitis, represent a potential risk for the PC insurgence [7]. Moreover, several studies demonstrated that hereditary germline or somatic mutations are responsible for tumor progression. Particularly, mutations in genes that are associated with cell death and proliferation as well as mutations in genes associated with telomerase shortening result in PC insurgence and metastasis [8,9]. Apart from the alteration of tumor suppressor genes and of the ones involved in the cell cycle regulation, cytokines have been shown to have a role in the malignant transformation [10]. Chronic inflammation, indeed, can lead to the production of several cytokines that activate different signaling pathways. This cascade results in the upregulation of other proinflammatory cytokines, such as interleukin-6 (IL-6) which affect the progression of the pancreatic cancer [11]. Among these pathways, Toll-like receptors (TLRs) seem to be activated during pancreas inflammation in response to damage-associated molecular patterns (DAMPs) [12]. Upon activation, TLRs, through different pathways, lead to the transcriptional factor NF-κB which supports the inflammatory microenvironment [13]. Several studies demonstrated that TLRs are upregulated in different neoplasia such as breast, lung and colon cancer where they are associated with a favorable or with a poor prognosis [14]. Recent reports demonstrated that TLRs are highly expressed also in pancreatic cancer where they are involved in the regulation of cancer physiology and therefore, they may represent a novel target for the cancer therapy [15,16,17]. In this review, we report the current knowledge about the role of TLRs in PC progression and we describe the compounds that may be implied in = PC treatment.

## 2. Toll-like Receptors

**Figure 1:**
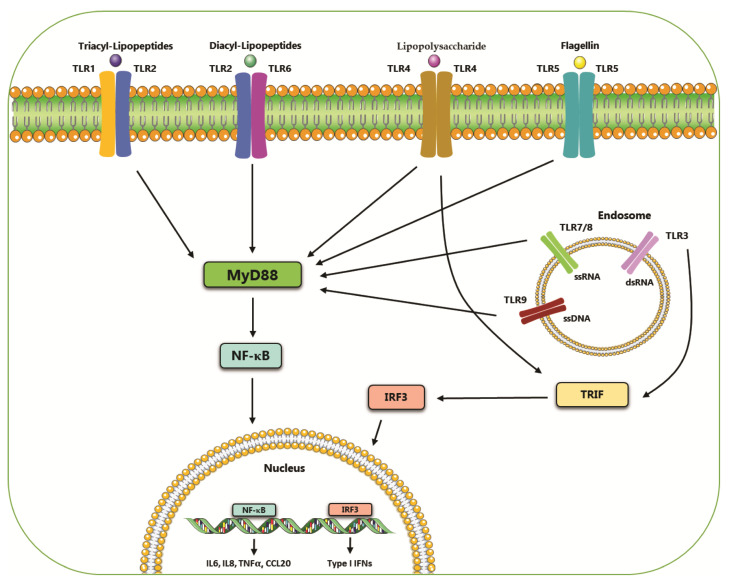
Localization and ligands of toll-like receptors (TLRs). The surface-expressed TLRs recognize bacterial compounds while the intracellular receptors recognize virus-associated nucleic acids. Almost all these receptors activate a signaling pathway that, through Myd88, leads to the activation of the transcriptional factor NF-κB. TLR3 and TLR4 activate a Myd88-independent signaling which culminates in the activation of transcriptional factor IRF3.

Toll-like receptors (TLRs) belong to the pattern recognition receptors (PRRs) family, which is involved in the activation of the innate immune response [18]. The PRRs family is able to recognize several pathogen-associated molecular patterns (PAMPs) deriving from pathogenic bacteria or fungi, viruses and protozoa [19]. TLRs consist of type I integral membrane glycoproteins with an extracellular N-terminal domain, that contains leucine-rich repeats (LRRs), and an intracellular C-terminal domain defined Toll/IL-1 receptor (TIR) domain [20,21]. TLRs family includes ten members: TLR1, TLR2, TLR4, TLR5, TLR6, TLR10, which are expressed extracellularly and TLR3, TLR7, TLR8, TLR9, that are expressed in the endosomes [22,23]. Furthermore, TLRs are classified according to the PAMPs that they are able to bind to: TLR1, TLR2, TLR4 and TLR6 recognize lipids, TLR5 and TLR10 detect proteins and TLR3, TLR7, TLR8 and TLR9 bind nucleic acids (Table 1) [24]. Upon stimulation, most of these receptors activate a signaling cascade that includes Myeloid differentiation primary response protein 88 (MyD88). This pathway, through the activation of several intermediates such as tumor necrosis factor (TNF) receptor-associated factor 6 (TRAF-6), IL-1R-associated kinases (IRAK) and mitogen-activated kinases, leads to the activation of the transcriptional factor NF-κB [25], a pleiotropic factor involved in the activation of pro-inflammatory genes [26] (Figure 1). In addition, TLRs, in particular TLR3 and TLR4, are able to activate a non-MyD88-dependent pathway that involves TIR-domain-containing adapter-inducing interferon-β (TRIF) protein and is responsible for the synthesis of interferon (IFN) and/or NF-κB activation [27,28]. Defects in the activation of TLRs result in the alteration of immune homeostasis that is sustained by the upregulation of NF-κB and the production of pro-inflammatory cytokines. This contributes to the development and progression of several diseases including cancer, diabetes type 1 and autoimmune diseases [29,30,31] (Figure 1).

## 3. Toll-like Receptor 1

TLR1 is expressed on the membrane of several lymphoid cell lines, including monocytes and lymphocytes and neuronal cells, such as CHP-212 and NT2-N [45,46]. This receptor is able to form a heterodimer with other TLRs acquiring the ability to recognize a broad range of antigens, such as bacterial proteins upon binding to TLR2 [47], and fungi upon binding to TLR6 [48]. Little is known about the role of this receptor in pancreatic cancer. Recently, a multivariate analysis has reported a positive correlation between the higher TLR1 expression and a better prognosis in pancreatic cancer patients who had received no post-operative adjuvant chemotherapy [49].

## 4. Toll-like Receptor 2

TLR2 expression profile varies among cell types with higher expression levels found on the plasma membrane of immune cells. Besides its role in inflammatory diseases, TLR2 plays, also, an antitumor activity that is exerted by several mechanisms such as enhancement of T-cell immunity, induction of apoptosis in TLR2-positive tumors and enhancement of the innate immunity [50]. In pancreatic cancer, increased TLR2 expression has a controversial role in the regulation of the pathophysiology of this neoplasia (Table 2). In particular, in pancreatic cancer cells, upon the binding of HMGB1 (High mobility group box1) to TLR2, the PI3K/pAKT pathway is activated with subsequential induction of the epithelial-mesenchymal transition necessary for the metastatic phenotype [33,34]. Furthermore, previous reports also described a role of TLR2 in the maintenance of stemness in ovarian and breast cancer cells Lately, a recent study demonstrated that, in pancreatic cancer cells, the interaction HMGB1 with TLR2 leads to the activation of Wnt/β-catenin in CD133+ cancer cells and it is responsible for the activation of stem cell genes, such as *NANOG*, *OCT4* and *SOX2* [35]. Leppanen et al. further discussed TLR2 expression in the pancreatic intraepithelial neoplasia (PanIN), a precursor of pancreatic cancer. Particularly, TLR2 expression varies among the different grades of severity of these lesions with lower expression in PanIN1 and higher TLR2 expression in PanN3 [51].

## 5. Toll-like Receptor 3

TLR3 is an endosomal receptor expressed in monocytes and dendritic cells with the ability to recognize double-stranded RNAs. Upon stimulation, this receptor activates a signaling pathway that ends up either in the activation of NF-κB or in the interferon-beta (IFNβ) production upon IRF3 activation. Previous evidence demonstrated the interplay between TLR3 and Wnt5a signaling in pancreatic cancer (Table 2). Particularly, PC cells show high expression levels of TLR3 associated with increased cancer cell proliferation and with constitutive activation of the Wnt5a signaling [36]. However, despite TLR3 expression in PC cells, it is still unclear which role it plays in pancreatic cancer pathophysiology.

## 6. Toll-like Receptor 4

TLR4 is a surface receptor, expressed either as homodimer or heterodimer together with TLR6 on the membrane of many immune cells, that recognizes the lipopolysaccharide (LPS), the major component of Gram-negative bacteria. Upon stimulation, TLR4 activates a downstream cascade which involves several adaptor molecules and culminates in the activation of the transcription factor NF-κB [62,63,64]. TLR4 expression is linked to several diseases. It has been reported that high TLR4 activation, upon LPS stimulation, is involved in the alteration of cytosolic Ca^2+^ and in cell death promotion, thus contributing to Alzheimer’s disease pathogenesis [65,66]. Moreover, PAMPs-induced TLR4 activation plays also a crucial role in inflammatory skin diseases [67,68]. Particularly, TLR4 stimulation activates a signaling that, through the recruitment of members of the CBM complex, leads to the activation of NF-κB-induced genes necessary for the maintenance of the inflammatory state [69,70]. Furthermore, TLR4 activation is involved in the promotion of several cancers, such as cervical [71], colorectal [72], and prostate cancer [73]. TLR4 upregulation has been found also in pancreatic cancer where it plays a central role in tumor progression. It has been demonstrated that stromal leukocytes from pancreatic cancer patients show high TLR4 expression levels. These data were confirmed by in vivo experiments in which KRAS mutated mice show upregulated levels of TLR4 both in stromal and epithelial cells while, on the other hand, TLR4^−/−^ mice had a reduction in tumor growth. Moreover, the high expression of TLR4 results in the activation of several NF-κB-induced genes, such as matrix metalloproteinases 2 and 9 (MMP2 and MMP9). Previous evidence reported that the proteolytic activity of these metalloproteinases is increased in pancreatic cancer cells co-cultured with M2-polarized macrophages in which the epithelial-mesenchymal transition (EMT) program is activated by TLR4/IL10 signaling pathway [52].

TLR4 upregulation is also involved in pancreatic cancer angiogenesis. It is well known that hypoxia upregulates different pro-angiogenic pathways that promote vessel growth [53]. Among these, the induction of TLR4 receptor by hypoxia-inducible transcription factor 1 alpha (HIF-1α) may facilitate pancreatic cancer growth as demonstrated in vitro by the exposure of PANC1 cells to hypoxic stress. Moreover, the regulation of TLR4 mediated by HIF-1α has been confirmed by knockdown experiments in which HIF-1α depletion is associated with inhibition of hypoxia-induced TLR4 overexpression and to pancreatic cancer regression [39].

Recently, a new role for TLR4 in pancreatic cancer progression has been reported. Specifically, the stimulation of TLR4 and CAP1 receptors with Resistin, a hormone released by macrophages in the cancer microenvironment, activates the STAT3 pathway that confers to pancreatic cancer cells the ability to resist cancer therapy [54]. Lately, Lanki et al. described a positive correlation for TLR2 and TLR4 in pancreatic cancer regression. In particular, they showed that the expression of these TLRs correlates with a favorable prognosis in patients with small tumor size and lymph-node-negative disease [15].

However, further investigation should be performed to shed light on the role of these receptors in pancreatic cancer pathogenesis.

## 7. Toll-like Receptor 5

TLR5 is expressed on the surface of several cell lines, such as adipocytes, leukocytes, intestinal and lung epithelial cells and in some tumor cells. Upon stimulation with flagellin, from mobile bacteria, TLR5 activates a signaling pathway that regulates several processes including insulin resistance, maintenance of lung and intestinal homeostasis and cancer [74]. Little is known about the role of TLR5 in pancreatic cancer. However, it has recently been shown that ligands within the gut microbiome of pancreatic cancer patients are recognized by TLR5, which, upon interaction with TLR2, activates a signaling cascade that leads to the cancer growth enhancement and to the suppression of innate and adaptive immune response [55,56].

Furthermore, in other cancers, polymorphisms in TLR5 receptor drive a differential cancer-promoting inflammation that is responsible for different clinical outcomes of cancer patients. Indeed, in breast cancer, deficiency in TLR5 activity is associated with an increased cancer progression while, on the other hand, TLR5 upregulation in ovarian cancer has a negative effect on long-term survival [75].

## 8. Toll-like Receptor 7

TLR7 is an endosomal receptor whose activation leads to an immune response upon the recognition of viral ssRNA. This sensor is predominantly expressed in specific immune cells such as plasmacytoid dendritic cells (pDCs) and B cells and in lower levels in keratinocytes, hepatocytes and epithelial cells [76]. The role of TLR7 and its agonists in cancer insurgence and progression is currently still controversial and further investigation is required to shed light on TLR7 function in cancer pathogenesis. Indeed, in renal and bladder cancer, upon activation with selective agonists, TLR7 shows anti-proliferative and apoptosis-inducing effects that result in reduced tumorigenesis. On the other hand, an opposite role has been described for TLR7 agonists in cell chronic lymphocytic leukemia cells where TLR7 stimulation acts as a pro-survival factor [40].

In pancreatic cancer, TLR7 expression was markedly increased in the progression from PanINs to metastatic cancer both in humans and mice. In particular, stimulation of TLR7 receptor increases cancer progression through the downregulation of cell cycle members such as cyclin D1 and p16, the downregulation of phosphatase and tensin homolog deleted on chromosome (PTEN) and the upregulation of p27, p53, p21, cyclin B1, PPARγ and TGF-β [16]. Furthermore, in epithelial cells, TLR7 stimulation leads to multiple signaling activation, such as STAT3, Notch, MAP kinase and NF-κB in epithelial cells, confirming that pancreatic cancer is driven by stromal inflammation [16].

Recent studies described a stage-dependent expression of TLR7 and TLR8 in the ductal pancreatic cancer [41]. A functional analysis performed in pancreatic cancer cells, PANC1, showed that increased chronic inflammation due to higher NF-κB activation and COX-2 expression is responsible for the increased cancer cell proliferation and chemoresistance [41]. Furthermore, it has been demonstrated that TLR7 and TLR8 activation is linked to Notch-2 receptor stimulation which results in the insurgence of chemoresistance against 5-fluorouracil in PANC-1 cells 10 [57].

## 9. Toll-like Receptor 9

Like TLR3 and TLR7, TLR9 is an endosomal receptor that recognizes unmethylated CpG-DNA and viral DNA [77,78]. This receptor shows a different expression profile with high expression in immune cells, such as plasmacytoid dendritic cells (pDCs), monocytes/macrophages, T and B cells and in non-immune cells, such as respiratory epithelial cells and keratinocytes [79,80].

Furthermore, TLR9 expression is associated with unfavorable prognosis in several cancers including squamous cell carcinoma of the tongue, esophageal adenocarcinoma and prostate cancer [81,82,83] and with a favorable prognosis in renal cell carcinoma and in triple-negative breast cancer [84,85]. It has been reported that, in pancreatic cancer, high TLR9 expression is associated with the increase of patient survival up to 15 months [58]. In particular, the stimulation of TLR9 plays an inhibitory role in pancreatic cancer cell proliferation. This effect was confirmed by in vivo studies in which the stimulation of TLR9 receptor with a synthetic agonist leads to pancreatic cancer regression through the inhibition of cancer cells growth and the enhancement of the immune response [43,59,60]. On the other hand, the activation of TLR9 is responsible for the fibrotic phenotype of pancreatic stellate cells and for the promotion of epithelial cell proliferation, and this confers to TLR9 a cancer-promoting role in the pancreatic cancer [44]. Recently, it has been reported that TLR9 expression is associated with microenvironmental pathogens. Indeed, during the pancreatic transformation, the gut microbiome may act as a source of TLR9 ligands which are responsible for the increased expression of TLR9 [61]. Nevertheless, further evidence is required to better define the association between TLR9 prognostic effect and microenvironment pathogens.

## 10. TLRs Agonists

TLR agonists play a fundamental role in activating innate and adaptive immune responses, and are, therefore, considered to be potent immunomodulators. For this reason, their use is being explored in cancer treatment both as monotherapy or in combined therapeutic strategies [86]. It has been suggested that TLR agonists could enhance the sensitivity of cancer cells to chemotherapy, radiation, and immunotherapy as well as improve the immunogenicity of ex vivo dendritic cells (DC) vaccines [87]. While the use of TLR agonists for the prevention and/or treatment of several disorders is a promising approach, many of these compounds were withdrawn from further studies due to limited efficacy or for the presence of side effects [88,89]. In this section, we will discuss the use of TLR agonists in PC treatment, while a more comprehensive list of the main TLR agonists is present in Table 3.

Macrophage activating lipopeptide-2 (MALP-2) is a synthetic lipopeptide capable of inducing immune responses through TLR2 and TLR6 activation [90,91]. It has been shown that MALP-2 can reduce tumor growth, prolong survival and increase the efficacy of gemcitabine treatments in murine in vivo models of pancreatic adenocarcinoma (PDAC) [92].

Since the cell line (Panc-2) used to generate those data do not express TLR2, the authors hypothesized that the MALP-2 effect could be through CD8+ lymphocytes and NK cells. Indeed, it has been shown that MALP-2 can activate DC through TLR2/TLR6 [93], even if, a later study described that this lipopeptide is unable to induce DC-TLR2 mediated NK cell activation [94]. However, results of a phase I/II trial that include MALP-2 treatment were encouraging [95]. In particular, ten patients who underwent laparotomy with incomplete or no resection of pancreatic adenocarcinoma were treated intraoperatively with MALP-2. The trial showed that the drug was well tolerated with a median survival of 9.3 months and mean survival of 17.1 months. An increase in the expression of co-stimulatory molecules on lymphocytes, and cytotoxic T and NK cells infiltrating the tumor was observed, leading to the hypothesis that MALP-2 could increase the activation of both the innate and the adaptive immune system. Currently, no other clinical trials were reported.

Another TLR2 agonist is protein-bound polysaccharide-K (PSK) that has been shown to enhance apoptosis and inhibit tumor growth in human PDAC cell lines [96]. More recently, evidences showed that PSK also inhibits hedgehog signaling by downregulating the expression of mastermind-like 3 (MAML3) and recombination signal binding protein for the immunoglobulin-kappa-J region (RBPJ) under hypoxia, inhibiting Smoothened (SMO) transcription and, thus, suppressing the malignant phenotype of PDAC cells [97,98].

Pancreatic adenocarcinoma upregulated factor (PAUF) is a protein overexpressed in the human PDAC [99] and other cancers [100,101,102]. PAUF promotes metastasis by regulating the TLR/CXCR4 activation [103].

PAUF has been shown to be an endogenous ligand for TLR2 and TLR4, it activates the canonical signaling pathways of TLR2-tumor progression locus 2 (TPL2)/mitogen-activated ERK kinase (MEK)/extracellular signal-regulated kinase (ERK), but it fails to mediate TLR2-induced NF-κB activation [103]. Recently, it has been demonstrated that PAUF can enhance the antigen-specific CD8+ T cell antitumor immunity of DC vaccines [104].

Polycytidylic acid (Poly I:C) is a TLR3 agonist capable of enhancing the cytotoxic activity and granzyme A/B production of γδ T cells in vitro [38]. Cell lines from pancreatic adenocarcinomas, squamous cell carcinomas of head and neck and lung carcinomas expressing TLR3 and TLR7 were pretreated with Poly I: C before co-culture with γδ T cells. The authors hypothesized that the observed effect on cytotoxicity was a consequence of the upregulation of CD54 on the tumor cells, and its consequent interaction with CD11a/CD18 expressed on γδ T cells.

Although it was also reported that Poly I:C can accelerate pancreatic carcinogenesis in KRAS-mutated mice [16] more recent evidence still supports its possible use as an adjuvant [37,105,106].

Metzger et al. provided evidence that Poly I:C can functionally reprogram myeloid-derived suppressor cells (MDSC) in orthotopically implanted Kras^G12D^ p53^fl/R172H^ Ptf1a-Cre (KPC) pancreatic tumors. Whole transcriptomic analysis of MDSC populations showed an IFN pathway-enriched gene signature together with a shift from an M2/G2- towards an M1/G1-polarized phenotype. The authors showed that the suppressive phenotype is promoted by IFN receptor 1 (IFNAR1), however, it is not clear if the effect of Poly I:C is mainly through TLR3 or RIG-I-like helicases (RLH) [107].

The effect of Poly I:C on macrophage polarization could make this compound attractive in combination with hypomethylating agents and immunotherapy.

It has been recently shown, using the KPC mouse model (Kras^LSL.G12D/+^; P53^LSL.R172H/+^; Pdx1-Cre^tg/+^), that low dose treatment with the hypomethylating drug decitabine (DAC) can increase the efficacy of immune checkpoint inhibitors (ICI) therapy. However, the authors also reported an increase of M2 macrophages, following DAC treatment, that are predicted to antagonize ICI antitumor effects [108]. Therefore, it is tempting to speculate that adding Poly I:C to the sequential therapy DAC+ICI could further improve the efficacy of this combination by promoting the M1 polarization of TAMs.

Finally, administration of a formulation of Poly I:C with polyethylenimine ([Poly I:C]^PEI^) induced apoptosis in PDAC cells but not in normal pancreatic epithelial cells [109]. Specifically, [Poly I:C]^PEI^, on one hand, repressed XIAP and survivin expression and, on the other hand, it is responsible for the immune response activation through MDA-5, RIG-I and NOXA induction and inhibition of AKT phosphorylation. In vivo administration of [Poly I:C]^PEI^ inhibited tumor growth via AKT-mediated XIAP degradation in both subcutaneous and quasi-orthotopic models of PDAC [107].

Phenylmethimazole (C10) is a derivative of methimazole with anti-inflammatory properties. Specifically, its inhibitory effect on TLR3 leads to suppression of the dsRNA induced, TLR3-mediated IRF3/IFN-pathway. The administration of C10 was effective in inhibiting growth and migration of cancer cell lines, as well as in inhibiting tumor growth in vivo in nude or severe combined immunodeficient mice both for human pancreatic cancer and malignant melanoma [36]. Several studies described a negative role of the TLR7 agonist, gardiquimod, in pancreatic cancer progression. In particular, the stimulation with this agonist inhibits cell proliferation and activates the apoptotic program through the downregulation of B-cell lymphoma 2 (BCL2), cyclin B1 and cyclin E and through the upregulation of B-cell-associated X protein. Furthermore, upon stimulation with gardiquimod, TLR7 induces the expression of anticancer genes such as PTEN and tissue inhibitor of metalloproteinase 1 (TIMP-1) and it downregulates the expression of VEGF in BxPC-3 pancreatic cancer cells [42]. Another reported TLR7/8 agonist is Resiquimod (R848), which plays a role in the remodeling of pancreatic cancer immune microenvironment and is responsible for the activation of an anti-tumor response. In particular, R848 exerts an anti-cancer activity through the increasing of CD8+ T-cell infiltration, the formation of tertiary lymphoid structures and decreased Treg concentration, all events associated with a better prognosis in the human neoplasia [110].

Recently, a polysaccharide isolated from *Strongylocentrotus nudus* eggs (SEP) has been shown to inhibit pancreatic cancer growth through TLR4/MAPKs/NF-κB pathway signals and activate NK cells in vitro and in vivo. Moreover, the same compound also increased gemcitabine anti-tumor activity by up-regulating NKG2D/MICA while reducing its side effects through the suppression of ROS release in vitro and in vivo [111]. Interestingly, these findings seem to contradict previous reports suggesting that TLR4 inhibition by triptolide can enhance the sensitivity of pancreatic cancer cells to gemcitabine by inhibiting the TLR4/NF-κB signaling [112]. These discrepancies although not totally unexpected in pre-clinical models, highlight the complexity of successfully translating TLR-targeting compounds to the clinic.

Similar to TLR3 and TLR7, TLR9 is expressed on endosomal membranes of several immune cells [113], and it has been linked to acute pancreatitis and cancer [12,114].

Synthetic TLR9 agonists are oligodeoxynucleotides containing immunostimulatory CpG motifs (CpGODNs) and are used as vaccine adjuvants or as antiallergic agents [115].

The addition of low-dose CpGODNs targeting TLR9 to a vaccine based on immune stimulatory complexes (ISCOM) inhibited the tumor immune evasion in an orthotopic model of pancreatic carcinoma inducing an effective CTL-mediated tumor cell killing and prolonging mice survival [59]. Moreover, CpG-ODNs treatment showed synergy in combination with gemcitabine in an orthotopic human pancreatic carcinoma xenograft mouse model, reducing metastasis and overall survival compared with monotherapy alone [60]. Immunomodulatory nucleotides (IMO) are second-generation CpG-ODNs with higher metabolic stability. It has been shown that IMO administration can synergize with the anti-EGFR monoclonal antibody cetuximab by interfering with EGFR-dependent signaling both in vitro and in vivo systems of pancreatic carcinoma [116,117].

Another CpG-ODN (ODN2216) has been reported to be able to reduce proliferation and migration ability in PANC-1 cells in vitro [43]. Additionally, more recently, intratumoral injection of IMO-2155 has been shown to trigger a potent immune reaction, especially in combination with systemic anti-PD1 therapy in pancreatic cancer orthotopic model [118]. The authors hypothesize that IMO-2125 local treatment recruited antigen-presenting cells (APCs) into the tumor thus leading to a more effective immune response [118].

Currently, for pancreatic cancer, combination therapy including TLR9-activating CpGs is being evaluated in two clinical trials (Clinicaltrials.gov accessed on 28 October 2021: # NCT04612530 and # NCT04050085). At this time both studies are still recruiting and no result has been posted. However, they will help to shed light on whether TLR9 agonists could represent a valid strategy to potentiate immunotherapy as well as chemotherapy in PC.

## 11. TLRs Antagonists

TLR antagonists are compounds able to reduce or inhibit activation of TLRs signaling, therefore acting as modulators of the native immunity.

They can generally be divided into two families: direct and indirect TLR antagonists.

Direct TLR antagonists are mostly compounds that competitively bind to the TLRs but fail to induce the conformational change necessary for the signal propagation. On the other hand, indirect TLR antagonists block TLR-associated signaling without competing with their ligands [145,146].

The potential use of TLRs inhibitors in the treatment of human cancers has been evaluated in both pre-clinical and clinical studies.

Given the importance of the TLR4 pathway in cancer progression, it’s not surprising that currently most of the inhibitors developed are directed against TLR4 (Table 4).

E5564 (also known as Eritoran) is a structural analog of the lipid A portion of LPS which competitively binds to TLR4/MD-2, thus resulting in inhibition of LPS-induced inflammatory responses. Although originally developed for the treatment of severe sepsis [147] there is evidence supporting its use in the cancer therapy [147]. In 2016, in fact, Deguchi et al. found that Eritoran inhibited lung cancer progression in vivo, likely due to reduced tumor angiogenesis, lower levels of TAMs and CD11b^+^Ly6C^++^Ly6G^–^ myeloid-derived cells infiltration with a consequent increase in CD8^+^ T-cell tumor infiltration [148]. Moreover, Kuo et al. showed that treatment with Eritoran in murine models of colorectal carcinoma was able to inhibit the progression of bacterial LPS-induced colon cancer through induction of CD14/Src/PKCζ-mediated apoptosis and the blockade of TLR4-dependent proliferation [149].

TAK-242 (also known as Resatorvid) is a small molecule inhibitor developed by Takeda. Its inhibitory effect is a consequence of the selective binding to TLR4 TIR-domain, therefore preventing its interaction TRAM or TIRAP [150].

Reports from different groups show this compound as negatively affecting proliferation and invasiveness of different cancer cell lines while enhancing the anti-cancer effects of chemotherapeutic agents [151,152,153,154]. Moreover, topical TAK-242 administration was shown to suppress solar UV-induced skin tumorigenesis in SHK-1 mice antagonizing chronic UV-induced inflammatory signaling [155].

Surfactant protein A-derived (SPA4) is a peptide inhibitor derived from the TLR4-interacting region of the SP-A [156]. It has been shown that it can inhibit LPS-stimulated inflammatory responses, migration and invasion of colon cancer SW480 [157]. Similar results were also reported in the lung cancer [158,159].

CRX526 (also known as an aminoalkyl-glucosaminide-phosphate) is a synthetic molecule that mimics the active component of LPS that binds to TLR4 (lipid A) [160,161]. CRX-526 was reported to significantly reduce the tumor volume of colon cancer xenograft mice models likely through the suppression of the TLR4/NF-κB p65 axis [162].

CX-01 is a heparin-derived polysaccharide with a low anticoagulant activity that targets the interaction of TLR4 and its ligand high mobility group 1 protein (HMGB1) [163].

The use of this TLR4 inhibitor has been evaluated in clinical trials for the treatment of refractory acute myeloid leukemia (AML) and myelodysplastic syndrome (MDS). Remarkably, it has shown promise in combination with chemotherapy or with epigenetic therapy (Azacitidine) [163,164].

CXC195 is an indirect TLR4 antagonist that inhibits the interactions of TLR4, MyD88 and NF-κB. It also inhibits the translocation of NF-κB to the nucleus, as well as its DNA binding activity. It has been shown that CXC195 can induce apoptosis and inhibits proliferation in cellular models of hepatocellular carcinoma cells [165,166] and bladder carcinoma [167].

## 12. Conclusions

Despite the progress made in translational research and therapy, pancreatic cancer still remains a lethal disease. Emerging evidence demonstrates that inflammation and pancreatic cancer are strongly associated. In this scenario, TLRs play a crucial role by activating the pro-inflammatory pathways responsible for the production of cytokines ad chemokines necessary to create a favorable microenvironment for tumor growth. Since these receptors play a dual role, as they activate pathways that lead to immunosuppressive cytokines production, the employments of TLR agonists and antagonists in cancer therapy may represent a potential strategy to improve the survival rate of patients with pancreatic cancer. Furthermore, a better comprehension of the molecular adaptors and their role in the TLRs signal transduction may help for the modulation of TLRs response in therapeutic treatments. Here we summarized the current knowledge about the implication of TLRs in PC, but despite the encouraging results, further studies are needed to better comprehend the molecular signature of pancreatic cancer and successfully employ TLRs modulation in a clinical setting.

## Figures and Tables

**Table 1:** Toll-like receptors expressed in human pancreatic cancer cell lines.

TLRs	Localization	PAMPs	Adaptor	Pancreatic Cancer Cell Line	Refs.
TLR1	Plasma membrane	Triacyl lipopeptides	Myd88/TIRAP	Not reported	[19,32]
TLR2	Plasma membrane	Lycolipids, lipoprotein, lipoteichoic acid, peptidoglycan, zymosan	Myd88/TIRAP	BxPC-3; MIA PaCa-2, MDA Panc-28, SU 8686, SW-1990, AsPC-1, Panc-1	[19,33,34,35]
TLR3	Endosome	Double-stranded RNA	TRIF	AsPC-1, Colo357, Panc-89, PancTu-1, Pt45P1	[36,37,38]
TLR4	Plasma membrane	Lipopolysaccharide (LPS), heat shock proteins	Myd88/TIRAP	AsPC-1, BxPC-3, CFPAC, MIA PaCa-2, MDA Panc-28, Panc-1, Sw-1990	[34,35,39]
TLR5	Plasma membrane	Flagellin	Myd88	Not reported	[32]
TLR6	Plasma membrane	Diacyl lipopeptides, lipoteichoic acid	Myd88/TIRAP	Not reported	[32]
TLR7	Endosome	Single-stranded RNA	Myd88	Colo357, MIA PaCa-2, MDA Panc-28, Panc-1, Sw-1990, Panc-89, PancTu-1, BxPC-3	[38,40,41,42]
TLR8	Endosome	Single-stranded RNA	Myd88	Panc-1	[41]
TLR9	Endosome	DNA (CpG)	Myd88	GER, MIA PaCa-2, MDA Panc-28, Panc-1, Sw-1990, T3M4	[34,43,44]
TLR10	Endosome	Unknown	Unknown	Not reported	[32]

**Table 2:** Association between TLRs expression and pancreatic cancer prognosis.

TLRs	Expression	Pancreatic Cancer Prognosis	Refs.
TLR1	High	Favorable	[49]
TLR2	High	Favorable	[15]
		Unfavorable	[33,34,35,51]
TLR3	High	Unfavorable	[36]
TLR4	High	Favorable	[15]
		Unfavorable	[39,52,53,54]
TLR5	High	Unfavorable	[55,56]
TLR7	High	Unfavorable	[16,41,57]
TLR8	High	Unfavorable	[41,57]
TLR9	High	Favorable	[43,58,59,60]
		Unfavorable	[44,61]

**Table 3:** List of agonists of Toll-like receptors.

Drug Name	Target	Drug Class	Refs.
Pam_3_CSK_4_	TLR1/2	Synthetic triacylated lipopeptide	[119]
MALP-2	TLR2/6	synthetic lipopeptide	[90,91]
PSK	TLR2	Protein-bound polysaccharide	[96]
PAUF	TLR2/4	Peptide	[103,120]
SMP-105	TLR2	Components of cell wall skeleton isolated from *Mycobacterium Bovis*	[121]
CBLB612	TLR2/6	Synthetic derivative of mycoplasma lipopeptide	[122]
Phenylmethimazole (C10)	TLR3	Methimazole derivative	[36]
Poly I:C	TLR3	Synthetic analog of viral dsRNA (polyinosinic-polycytidylic acid)	[123,124]
PolyICLC	TLR3	Polyinosinic-polycytidylic acid mixed with the stabilizers carboxymethylcellulose and polylysine	[125]
Poly-IC12U	TLR3	Poly I:C derivative with shorter half life and less toxicity	[126]
IPH 3102	TLR3	Synthetic dsRNA agent	[127]
ARNAX	TLR3	Synthetic DNA/RNA hybrid molecule	[128]
MPLA	TLR4	Lipid A derivative	[129]
OK-432	TLR4	Lyophilized mixture of group A *Streptococcus pyogenes*	[130]
AS04	TLR4	Combination of MPLA and aluminum salt	[131]
GLA-SE (G100)	TLR4	Glucopyranosyl lipid-A oil-in-water emulsion	[132]
CBLB502	TLR5	derivative of *Salmonella* flagellin	[133]
M-VM3 (Mobilan)	TLR5	Recombinant non-replicating adenovirus that directs expression of human Toll-like receptor 5 and of a flagellin derivative that acts as a selective agonist of TLR5	[134]
ssRNA40	TLR7	20-mer phosphorothioate protected single-stranded RNA oligonucleotide containing a GU-rich sequence	[16]
Gardiquimod	TLR7	Imidazoquinoline compound	[42]
Resiquimod (R848)	TLR7	Imidazoquinoline compound	[110]
Bistriazolyl	TLR7	Small Molecule	[135]
VTX1463	TLR8	Small Molecule	[136]
CpG-1826	TLR9	Oligodeoxynucleotide containing immunostimulatory CpG motifs	[122]
CpG-7909	TLR9	Oligodeoxynucleotide containing immunostimulatory CpG motifs	[137]
IMO-2155	TLR9	Oligodeoxynucleotide containing immunostimulatory CpG motifs	[138]
MGN1703	TLR9	Covalently closed natural DNA molecule	[139]
dSLIM	TLR9	MGN1703 derivative	[140]
SD-101	TLR9	Oligodeoxynucleotide containing immunostimulatory CpG motifs	[141]
KSK-CpG	TLR9	Oligodeoxynucleotide containing immunostimulatory CpG motifs	[142]
ODN2216	TLR9	Oligodeoxynucleotide containing immunostimulatory CpG motifs	[143]
ODN M362	TLR9	Oligodeoxynucleotide containing immunostimulatory CpG motifs	[144]

**Table 4:** List of antagonists of TLRs.

Drug Name	Target	Drug Class	Refs.
TAK-242 (Resatorvid)	TLR4	Small molecule inhibitor	[150]
CRX-526	TLR4	Synthetic lipopolysaccharide	[163]
CX-01	TLR4	heparin-derived olysaccharide	[164]
CXC195	TLR4	tetramethylpyrazine analogue	[165]
Eritoran (E5564)	TLR4	Synthetic lipopolysaccharide	[168]
Atractylenolide-1	TLR4	sesquiterpene compound	[169]
Triptolide	TLR4	diterpenoid epoxide	[170]
Paeonol	TLR4	Small molecule inhibitor	[171]
NI-0101	TLR4	Monoclonal antibody	[172]
Nalmefene (JKB-121)	TLR4	Small molecule inhibitor	[173]
Ibudilast (AV-411, N-166)	TLR4	Small molecule inhibitor	[174]
Polymyxin B (PMB)	TLR4	Cyclic polypeptide antibiotic	[175]
OPN305	TLR2	Monoclonal antibody	[176]
Hydroxychloroquine	TLR7,9	Quinolone	[177]

## Data Availability

Not applicable.
